# Four phases of a force transient emerge from a binary mechanical system

**DOI:** 10.1007/s10974-024-09674-8

**Published:** 2024-05-30

**Authors:** Josh E. Baker

**Affiliations:** Department of Pharmacology, University of Nevada, Reno School of Medicine, Reno, NV USA

**Keywords:** Muscle, Thermodynamic, Transient, Entropic Spring, Myosin Switches

## Abstract

Accurate models of muscle contraction are important for understanding both muscle performance and the therapeutics that enhance physiological function. However, models are only accurate and meaningful if they are consistent with physical laws. A single muscle fiber contains billions of randomly fluctuating atoms that on the spatial scale of a muscle fiber generate unidirectional force and power output. This thermal system is formally constrained by the laws of thermodynamics, and a recently developed thermodynamic model of muscle force generation provides qualitative descriptions of the muscle force-velocity relationship, muscle force generation, muscle force transients, and the thermodynamic work loop of muscle with a thermodynamic (not molecular) power stroke mechanism. To demonstrate the accuracy of this model requires that its outputs be quantitatively compared with experimentally observed muscle function. Here I show that a two-state thermodynamic model accurately describes the experimentally observed four-phase force transient response to both mechanical and chemical perturbations. This is the simplest possible model of one of the most complex characteristic signatures of muscle mechanics.

## Introduction

The molecular mechanism of muscle contraction is a force-generating switch [a discrete lever arm rotation induced by actin binding and gated by the release of inorganice phosphate (Huxley and Simmons 1971; Rayment et al. 1993; Finer et al. 1994; Baker et al. 1998, 2002)]. An ensemble of molecular switches (a binary mechanical system) is an entropic spring that shortens to perform work (Baker and Thomas 2000; Baker 2022). This is the thermodynamic power stroke mechanism of muscle contraction (Baker 2022). The energetics and mechanics of a thermodynamic power stroke are described by the Gibbs free energy equation (Baker 2022). In contrast, the energetics and mechanics of a Huxley-Hill molecular power stroke are described by Hooke’s law (Huxley 1957; Hill 1974). A thermodynamic power stroke mechanism cannot be defined by conventional Huxley-Hill molecular models because the system entropy and system mechanics of an entropic spring are not defined within individual myosin motors. Indeed, thermodynamics and Huxley-Hill represent mutually exclusive models of muscle contraction. While the former – consistent with the 2nd law of thermodynamics – takes into account the entropy of the muscle system, the latter does not (Baker 2023a, 2024).

**Fig. 1:**
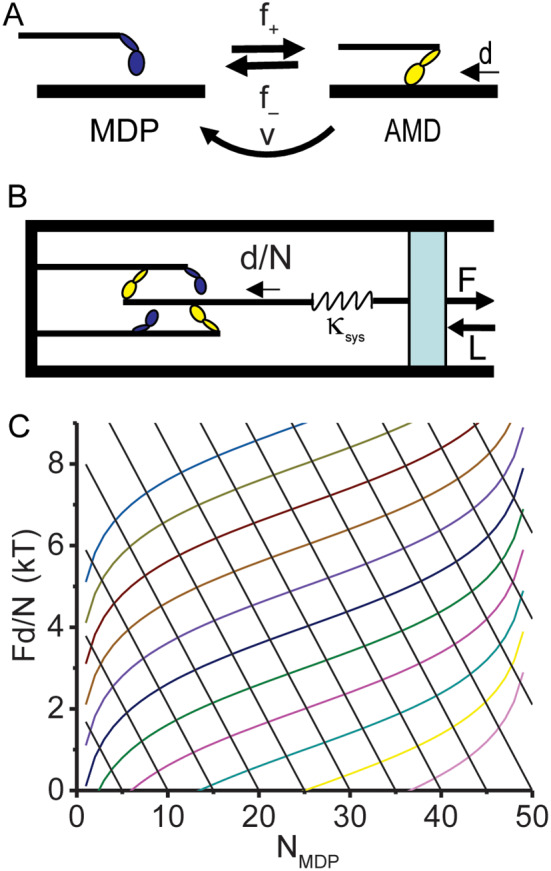
Binary Mechanical Model System. **A** A two state (MDP and AMD) scheme in which a myosin motor, M, with bound ADP, D, and inorganic phosphate, P_i_, undergoes a discrete conformational change upon binding actin, A, and releasing P_i_ to generate a displacement, *d*, at a rate *f*_*+*_. The reverse transition occurs at a rate *f*_*–*_. Through the actin-myosin-catalyzed ATP hydrolysis reaction, AMD is irreverbly transferred to MDP at the ATPase rate, *v*. **B** The laws of mechanics of a binary mechanical system can be described by a single system spring of stiffness *κ*_*sys*_ that on one end (right) describes the macroscopic mechanics (force, *F*, and length, *L*) of muscle and on the other end (left) describes the molecular force, *κ*_*sys*_*d*/*N*, generated with each myosin binding step. **C** Eq. 4 (colored curves) is plotted at ∆G^o^ increments of 1 kT, and Eq. 3 is plotted (black lines) at *N*_*MDP*_^*o*^ increments of 5

A thermodynamic model of muscle contraction was first proposed in 1938 by A.V. Hill (Hill 1938). We first observed ensembles of myosin motor switches in active skinned muscle in 1998 (Baker et al. 1998), and determined how this ensemble of switches is coupled to muscle force, *F*, one year later (Baker et al. 1999). The implication that a binary mechanical system is the mechanism for Hill’s thermodynamic muscle model was first proposed in 2000 (Baker and Thomas 2000), subsequently developed into the binary mechanical model of muscle force generation illustrated in Fig. 1 in 2022 (Baker 2022), and established as an entropic spring in 2023 (Baker 2022, 2023b). Single molecule mechanics and structural studies provide experimental support for a single molecular mechanism of muscle contraction: a force-generating myosin switch (Huxley and Simmons 1971; Rayment et al. 1993; Finer et al. 1994; Baker et al. 1998, 2002). Because a force-generating myosin switch is not a molecular power stroke (it does not generate power output on the time scale of muscle shortening), a molecular mechanism for muscle’s power stroke remains undefined and unobserved (Baker 2023a). In contrast, a thermodynamic power stroke (the shortening of an ensemble of molecular switches on the time scale of muscle shortening) is consistent with a force-generating myosin switch as the sole molecular mechanism of muscle contraction (Baker 2023a).

Recently, I developed the first thermodynamic model of muscle force generation and presented qualitative outputs for the muscle force-velocity relationship, force transients, work loops, and force generation (Baker 2022). However, to demonstrate the accuracy of this model, quantitative comparisons to experimental data are needed. We have shown that this model accurately describes the muscle force-velocity relationship (Baker and Thomas 2000). Here, I show that it accurately accounts for muscle force transients.

Transient force responses to rapid mechanical and chemical perturbations to isometric muscle have been well characterized with the goal of providing insights into fast kinetics and mechanics (Civan and Podolsky 1966; Huxley and Simmons 1971; Ford et al. 1977; Kawai and Halvorson 1991; Dantzig et al. 1992). In 1971, Huxley and Simmons showed (Huxley and Simmons 1971) that a rapid lengthening or shortening step of an isometric muscle fiber results in a four-phase transient force response. In 1992, Dantzig et al. (Dantzig et al. 1992) showed that a rapid chemical perturbation to a skinned isometric muscle fiber resulted in a three-phase transient force response. According to a molecular power stroke model, the mechanisms for transient force responses differ for chemical and mechanical perturbations (Dantzig et al. 1992) with a different molecular mechanism required for each transient phase (Huxley and Simmons 1971; Kawai and Halvorson 1991). Here I show that a single entropic spring accurately accounts for all phases of the muscle transient force response following both chemical and mechanical perturbations.

A thermodynamic muscle model describes the four phases of a force transient as follows: Phase 1 is a rapid change in system energy, *δE*, that perturbs the force, *F*, of an entropic spring relative to the equilibrium force, *F*_*o*_, by changing either *F* (mechanical perturbation) or *F*_*o*_ (chemical perturbation); Phase 2 occurs when the non-equilibrium entropic spring through force-generating steps equilibrates with a non-ergodic force, *aF*_*o*_; Phase 3 is a chemical relaxation toward the ergodic equilibrium force, *F*_*o*_; and Phase 4 is a return to the initial equilibrium force along the ergodic isotherm. These are the same four phases for both chemical and mechanical perturbations (phase 1 of a chemical perturbation, *δE*, does not involve a change in force). This analysis demonstrates that the simplest possible muscle model – a single entropic spring – accurately describes one of the most complex mechanical behaviors of muscle.

## Results

### Model summary

All simulations of muscle force transients herein are based on a recently published binary mechanical model of muscle force generation (Baker 2022) described in the methods section. Briefly, the model assumes that the molecular mechanism of muscle contraction is the force-generating molecular switch of a myosin motor (Fig. 1A) directly observed in single molecule mechanic studies (Huxley and Simmons 1971; Rayment et al. 1993; Finer et al. 1994; Baker et al. 1998, 2002). An ensemble of molecular switches is an entropic spring that shortens to perform work (Fig. 1B). Force is generated in an entropic spring through one of two thermodynamic processes. An isothermal stretch of an entropic spring (the right side of the spring in Fig. 1C) is described by the binding free energy equation (Eq. 4, plotted as colored curves in Fig. 1C), and adiabatic (isometric) displacements of the left side of the spring in Fig. 1C are generated by force-generating switches (Eq. 3, plotted as straight lines in Fig. 1C). Isometric force generation occurs when the right side of the spring in Fig. 1C is fixed, which I refer to as adiabatic because no shortening heat is lost. The time courses of the external force, *F*, and the number of motors, *N*_*MDP*_ and *N*_*AMD*_, in states MDP and AMD (myosin with bound ADP and phosphate, and actin-myosin with bound ADP, Fig. 1A) are described by two master equations (Eqs. 7 and 8) through which Eqs. 3 and 4 are coupled by rates, *f*_*+*_ and *f*_*–*_, (Fig. 1A) that are defined (Eq. 6) in terms of system energetics (Baker 2022).

The two master equations (Eqs. 7 and 8) describe the time course over which a non-equilibrium isometric entropic spring equilibrates at the ergodic (true equilibrium) isotherm at an equilibrium force, *F*_*o*_, defined by Eq. 4. However, there are many mechanisms that can prevent an entropic spring from reaching the ergodic isotherm, such as sequestration of myosin motors in inactive states, a stiff system in which a small number of myosin motors generate a large force, and work performed on or by internal elements that affect the total energy available for force generation. In these cases, the system equilibrates at a non-ergodic isotherm because myosin switches equilibrate with (are “damped” or “frustrated” by) internal factors as well as the external force. An ergodicity factor, *a*, describes both the fractional offset of the non-ergodic isotherm from the ergodic isotherm and a non-ergodic equilibrium force, *F* = *aF*_*o*_. The factor *a* can be thought of as the fraction of motors that equilibrate with the external force. If these internal damping factors are transient, the system eventually equilibrates at the ergodic isotherm at the rate at which internal damping is overcome, *b*.

### Simulations of ideal adiabatic force generation and isotherm stretch and shortening

**Fig. 2:**
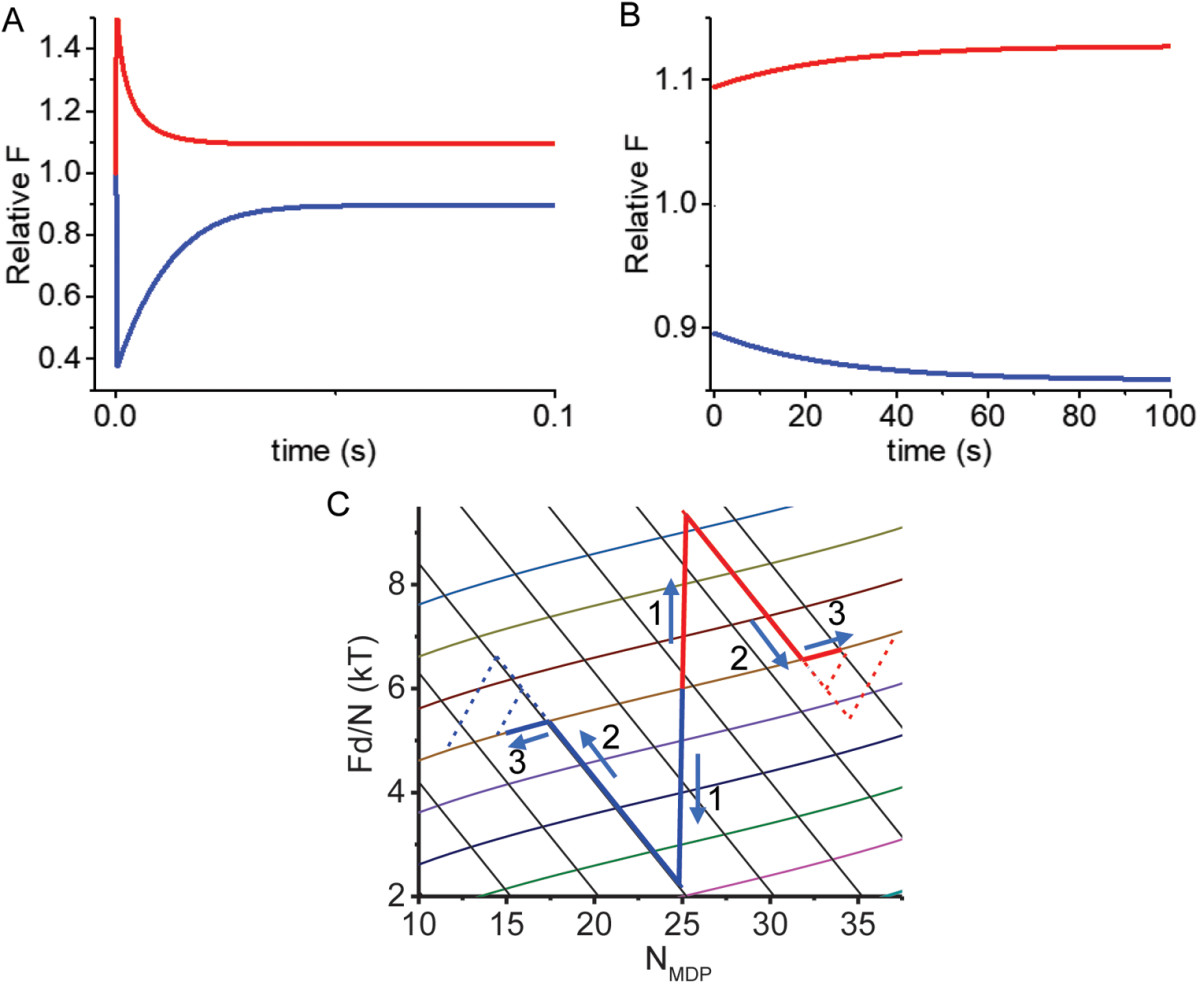
Simulated force transients following rapid length steps, ΔL, using Eqs. 7 and 8 and rate constants defined by Eq. 6. Parameters were initialized to values in Table 1, with *N*_*AMD*_ = *N*_*MDP*_ = 25 and *F*_*i*_ = 41.3 pN (Relative *F*_*i*_ = 1). **A** At t = 0, *F*_*i*_/κ_sys_ was both decreased by Δ*L* = 5.26 nm from a relative *F*_*i*_ of 1 to 0.36 (blue curve) and increased by Δ*L* = 4.74 nm from a relative *F*_*i*_ of 1 to 1.6 (red curve). Force transients initialized at each non-equilibrium *F* value were then simulated. **B** Slow changes in the length of the system spring initialized with equilibrated values for *F*, *N*_*AMD*_, *N*_*MDP*_ in panel A were simulated. An increase in length was simulated for equilibrated values following the length increase in panel A (red line), and a decrease in length was simulated for equilibrated values following the length decrease in panel A (blue line). **C** Values for *F* and *N*_*MDP*_ from simulations in panels A and B are replotted as *Fd*/*N* vs. *N*_*MDP*_ and overlaid with Eq. 4 (colored curves) and Eq. 3 (solid black lines). The simulations in panels A and B were repeated using values for *z* of 0.4 (low amplitude) and 0.8 (high amplitude) and replotted in panel C (dashed lines)

Figure 2 shows simulations of ideal adiabatic (Fig. 2A) and isothermal (Fig. 2B) processes. In Fig. 2A, an equilibrium force, *F*_*o*_ = –*N*∆G^o^/*d*, is rapidly changed by either a rapid increase (red line) or decrease (blue line) in the the force, ∆*F*, or length, ∆*L*, of the entropic spring, perturbing the system energy by *δE* = ∆*F*⋅*d/N* = *k*_*sys*_⋅∆*L*⋅*d/N* (phase 1). The two master equations (Eqs. 7 and 8) describe the time course over which the non-equilibrium isometric entropic spring equilibrates at the ergodic isotherm at an equilibrium force, *F*_*o*_, defined by Eq. 4 (phase 2). Starting with the parameters simulated at the end of phase 2 in Fig. 2A, in Fig. 2B the two master equations (Eqs. 7 and 8) describe the time course of the equilibrium entropic spring being slowly lengthened (red line) or shortened (blue line). The simulations in Fig. 2A and B (red and blue lines) are replotted in Fig. 2C (solid red and blue lines) as *F*⋅*d*/*N* versus *N*_*MDP*_ and are overlaid with Eqs. 3 and 4, showing that transient adiabatic and isothermal simulations are consistent with the thermodynamic relationships in Eqs. 3 and 4.

**Table 1:** Model Parameters. All values are consistent with experimental measurements in muscle and muscle myosin where rate constant references are from R temporaria and mechanical parameters references are from skeletal muscle. The parameter a_Go_ was determined from *f*_*+*_ ^o^ = exp[a_Go_⋅∆G^o^/kT] assuming *f*_*+*_ ^o^ = 40 s^–1^ and gives a value for *f*_*–*_^o^ = exp[–(1 – a_Go_)⋅∆G^o^/kT] of 0.1 s^–1^. All other model parameters are determined using first principles (equations derived herein)

Parameter	Description	Value
N	Number of myosin heads	50 (Stewart et al. 2021)
∆G^o^	Standard reaction free energy for actin-myosin binding induced switch	–6 kT (Baker and Thomas 2000)
d	Step size	8.7 nm (Guilford et al. 1997)
κ_sys_	Effective stiffness of system spring	5 pN/nm (Ford et al. 1981; Lewalle et al. 2008)
a	Ergodicity	0.3
b	Rate of mechanical equilibration	20 s^–1^
a_Go_	partition of standard reaction free energy	0.615 (Pertici et al. 2021)
a_Fd_	partition of work	0.3

Figure 2C shows that the change in system energy, *δE*, is fully recovered through adiabatic force generation (the simulations begin and end on the same isotherm). However if the lengthening of the entropic spring during phase 2 performs work in stretching internal elastic elements, a fraction, *z*, of *δE* is used to perform internal work on these elements, and the entropic spring approaches a non-ergodic isotherm that is offset by *z*⋅*δE* below the ergodic isotherm at a smaller force, *F* = (*F*_*o*_ – *z*⋅*δE*/*d*) = *aF*_*o*_, where *a* = 1 – (*z*⋅*δE*/*d*)/*F*_*o*_. Inversely, if the lengthening of the entropic spring during phase 2 shortens internal elastic elements, the work performed by these elements contributes energetically, *zδE*, to force generation, and *F* approaches a non-ergodic isotherm that is offset by *z*⋅*δE* above the ergodic isotherm (Fig. 2C, blue dashed lines along Eq. 3) at a higher force, *F* = (*F*_*o*_ + *z*⋅*δE*/*d*) = *aF*_*o*_, where *a* = 1 + (*z*⋅*δE*/*d*)/*F*_*o*_. In both cases, *zδE* is transient work performed with phase 2, which is eventually lost from the system, presumably at the rate of chemical relaxation, *b* = *f*_*+*_ ^o^ + *f*_*–*_^o^, when the entropic spring equilibrates at the ergodic isotherm (∆_r_G approaches zero and *a* approaches 1; Fig. 2C, blue dashed lines back to the ergodic isotherm).

### Simulations of adiabatic force generation and mechanical equilibration

**Fig. 3:**
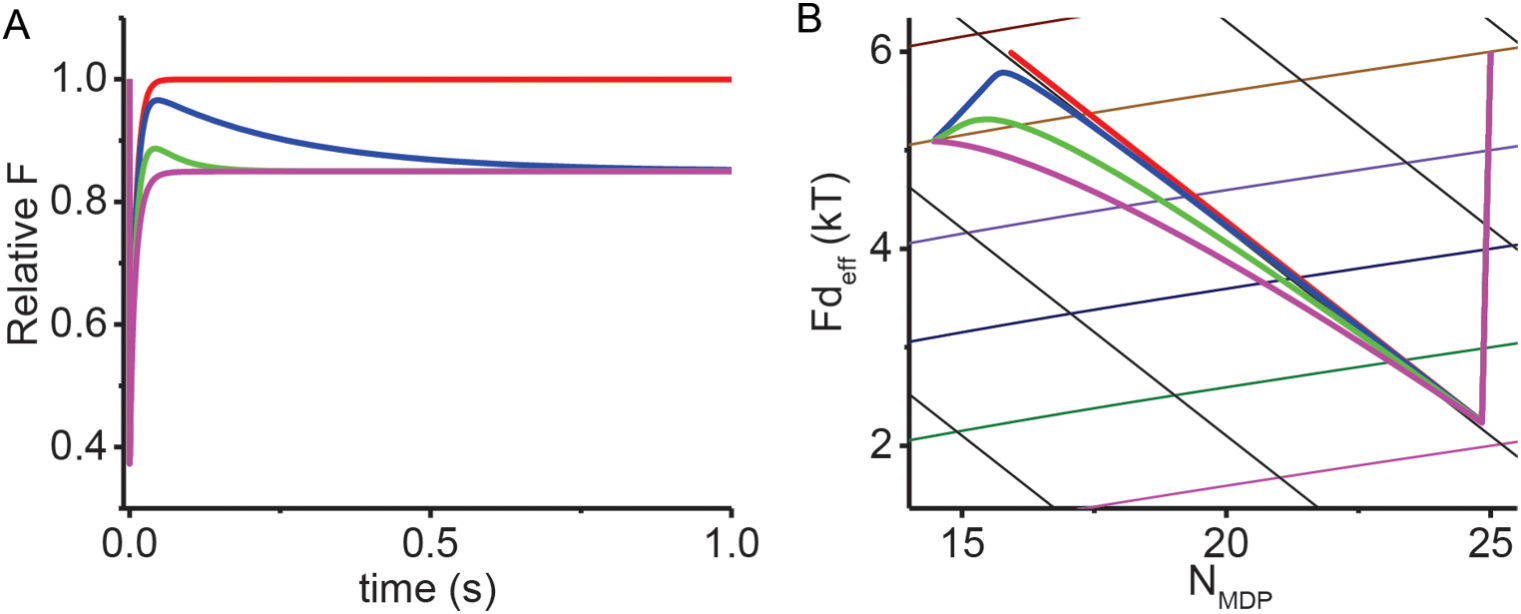
Simulated effects of mechanical equilibration rate, *b*, on force transients. **A** The same simulations performed in Fig. 2 following a rapid shortening step are repeated at *z* = 0.4 and different chemical relaxation rates, *b*, of 0 s^–1^ (red), 4 s^–1^ (blue), 20 s^–1^ (green), and 40 s^–1^ (magenta). **B** Values for *F* and *N*_*MDP*_ simulated in panel A are replotted as *Fd*/*N* vs. *N*_*MDP*_ and overlaid with Eq. 3 (solid black lines) and Eq. 4 (colored curves)

In Fig. 2C, phases 2 and 3 for all simulations are temporally separate because phase 3 is slow. However, when phase 3 occurs on a time scale comparable to that of phase 2 (Eq. 3), the two phases begin to merge. Figure 3A is the same simulation shown in Fig. 2C (*z* = 0.4) only here with rates, *b*, of 0, 4, 20, and 40 s^–1^. These simulations are replotted in Fig. 3B as *F*⋅*d*/*N* vs. *N*_*MDP*_. When *b* = 0, phase 2 follows Eq. 3 with no phase 3. With an increase in *b*, phase 2 deviates from Eq. 3 until when *b* equals the chemical relaxation rate (*f*_*+*_ ^o^ + *f*_*–*_^o^ = 40 s^–1^, Table 1), force recovery occurs with a single non-ideal phase intermediate that of Eq. 3 and Eq. 4.

The thermodynamic approach to modeling muscle contraction is top-down. That is, a thermodynamic model consists of a limited number of macroscopic parameters (Table 1) that are determined from fits of thermodynamic equations to experimental data (Eqs. 3 and 4). Molecular details are then inferred from fitted thermodynamic parameters. This is a familiar approach when inferring molecular mechanisms from measured changes in ∆G^o^ associated with protein mutations, post-translational modifications, small molecules, and changes in T, pH, and ionic strength. Similarly, the thermodynamic parameter *a* must be determined before molecular mechanisms can be inferred.

In muscle, the amplitude of phase 2 force generation following a rapid shortening step often overshoots isometric force, *F*_*o*_, demonstrating experimentally that *a >* 1. As described above, one possible mechanism is that a strained internal elastic element shortens during phase 2, energetically contributing to force generation and resulting in force that exceeds the ergodic force, *F*_*o*_. In muscle, titin is a large, passive elastic element (Kellermayer et al. 1997) that contributes to muscle force, and a decrease in length of a strained titin molecule during phase 2 would contribute to the free energy available for force generation, resulting in an non-ergodic equilibrium force, *F*, that exeeeds the equilibrium force, *F*_*o*_.

Experimentally, a rapid change in the length of steady state isometric muscle has been shown to elicit a multi-phasic force response. This was first observed in whole muscle (Gasser and Hill 1924; Jewell and Wilkie 1958) and subsequently observed with improved time resolution in single muscle fibers (Huxley and Simmons 1971; Ford et al. 1977). In 1974, Huxley and Simmons observed that when the length of an isometric muscle fiber was rapidly shortened or lengthened the force response occurs in four phases, resembling the four thermodynamic processes described above. Here, I compare simulations of these chemical thermodynamic processes with force transients observed by Huxley and Simmons.

**Fig. 4:**
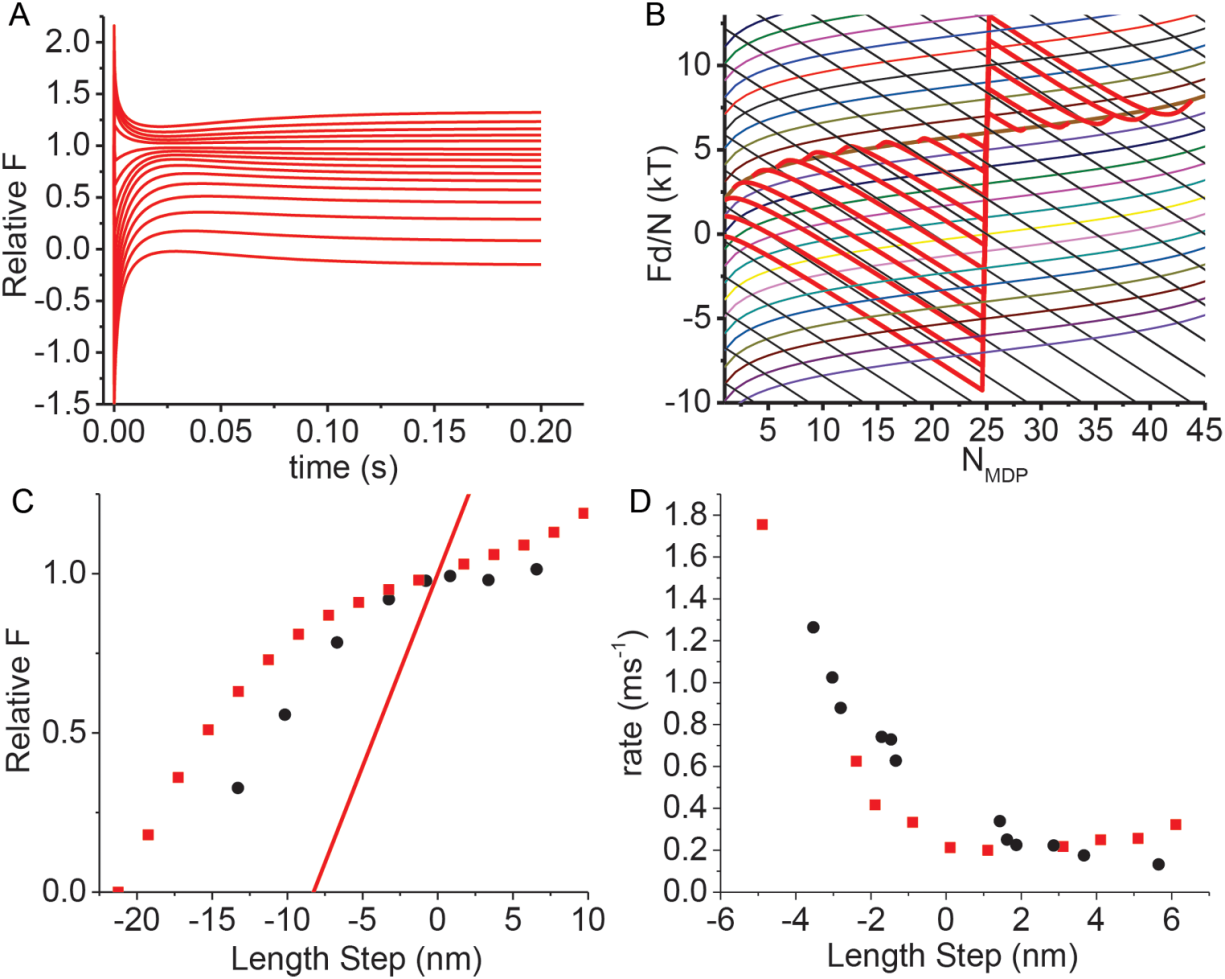
Simulated force transients following length steps, ΔL, using Eqs. 7 and 8 and rate constants defined by Eq. 6. Parameters were initialized to values in Table 1, with *N*_*AMD*_ = *N*_*MDP*_ = 25, *z* = 0.4, *b* = 20 s^–1^ and *F*_*i*_ = 41.3 pN (Relative *F*_*i*_ = 1). **A** At t = 0, *F*_*i*_/*κ*_*sys*_ was increased or decreased by different Δ*L* values followed by simulation as in Fig. 2A (red lines). **B** Values for *F* and *N*_*M*_ simulated in panel A are replotted as *Fd*/*N* vs. *N*_*M*_ (red lines) and overlaid with Eq. 3 (solid black lines) and Eq.  4 (colored curves). **C** Phase 2 amplitudes were determined from simulations in panel B and plotted (red squares) for different length steps, Δ*L*, and overlayed with experimental data (black circles) digitized from Huxley and Simmons (Huxley and Simmons 1971a). The force, *F*, immediately following the length step, Δ*L*, (Phase 1 amplitude) is *F* = *F*_*i*_ – Δ*L*⋅*k*_*sys*_ and is plotted (red line). (D) Phase 2 rates were determined from simulated transients in panel B as 1/*t*_*½*_, plotted (red squares) for different Δ*L*, and overlayed with experimental data (black circles) digitized from Huxley and Simmons (Huxley and Simmons 1971a)

Figure 4A shows simulated transient responses to length steps, Δ*L*, of different amplitudes, which are replotted as *F* vs. *N*_*MDP*_ in Fig. 4B. The transient responses are non-exponential, and so phase 2 rates were determined from 1/*t*_*½*_ of the simulated phase 2 where *t*_*½*_ is the time at which the force reaches ½ the sum of the maximum phase 1 and phase 3 force. Figure 4C is a plot of simulated phase 2 rates for different length steps overlaid with experimental data obtained from R temporaria muscle at 2° C (Huxley and Simmons 1971).

Figure 4D is a plot of the simulated phase 2 amplitudes for different length steps, Δ*L*, overlaid with experimental data obtained from R temporaria muscle at 2° C (Huxley and Simmons 1971). The mechanism underlying the Δ*L* dependence of the phase 2 amplitude is geometrically evident from Fig. 4B. Specifically, larger shortening steps, Δ*L*, require larger increases in *N*_*AMD*_ to equilibrate with the ΔG^o^ isotherm, which decreases the phase 2 amplitude by kT⋅ln(*N*_*AMD*_/*N*_*MDP*_) along the isotherm until at sufficiently large Δ*L*, *N*_*AMD*_ approaches *N*, and *F* falls off the isotherm, reaching *F* = 0 when *N*_*MDP*_^o^ = 0 (Eq. 4).

Force transients have also been measured in muscle following rapid chemical perturbations. For example, Dantzig et al. (Dantzig et al. 1992) showed in skinned muscle fibers that a rapid increase in [P_i_] upon photo-release of caged-P_i_ results in a multi-phasic force response. In these experiments, different final phosphate concentrations, [P_i_]_f_, were achieved through a combination of varying both initial concentrations, [P_i_]_i_, and the amount of caged-P_i_ photo-released. Using an analysis based on the molecular mechanic formalism, they conclude that the initial force response is different from the phase 2 response in a length step experiment and requires even more states (Kawai and Halvorson 1991; Dantzig et al. 1992). Here, I compare the four phases of a mechanical transient in a binary mechanical system with the mechanical response to [P_i_] jumps observed by Dantzig et al.

**Fig. 5:**
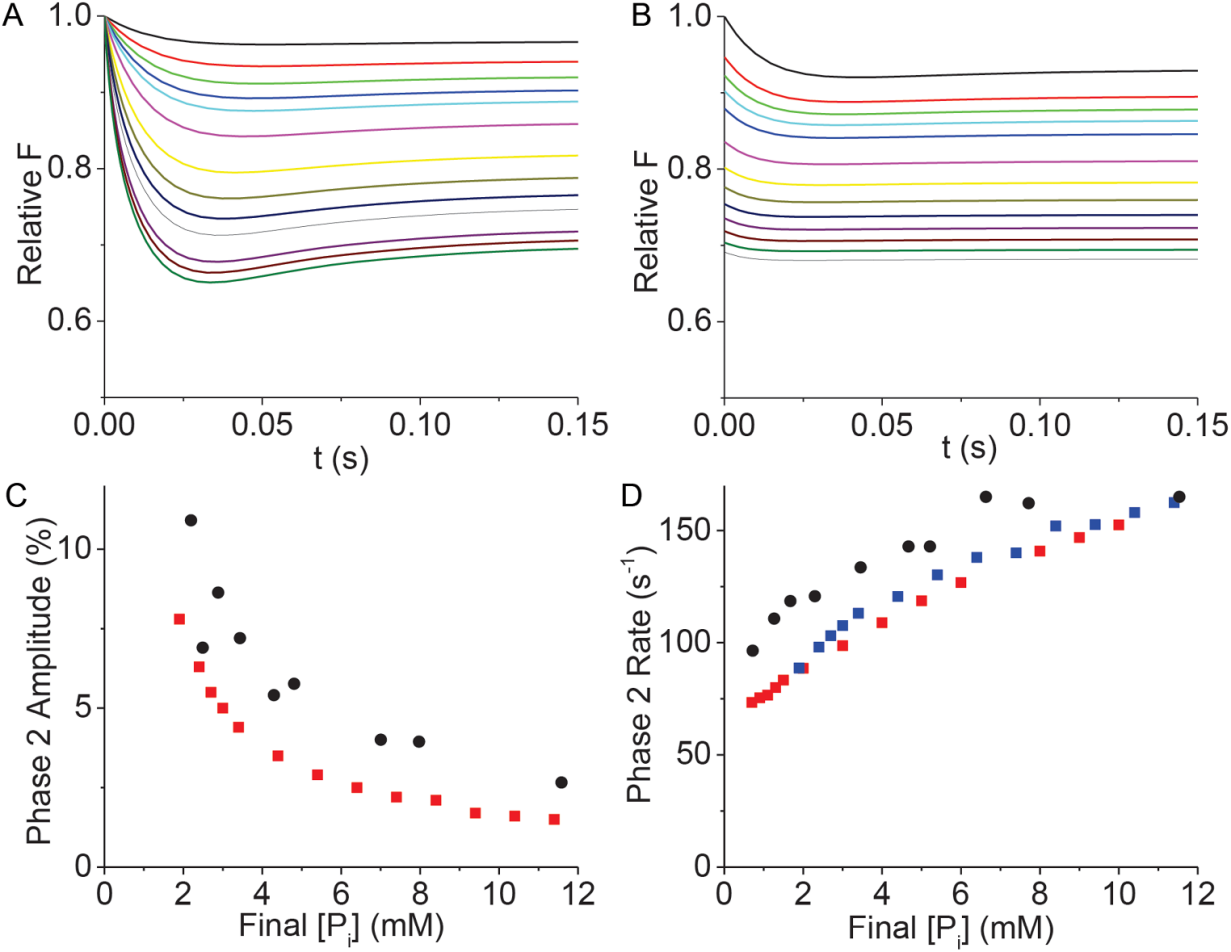
Simulated force transients following rapid increases in [P_i_], using Eqs. 7 and 8 and (Dantzig et al. 1992) rate constants defined by Eq. 6. Parameters were initialized to values in Table 1 (except *f_*° = 0.01 s^− 1^), *N*_*AMD*_ = *N*_*MDP*_ = 25, *F*_*i*_ = 137.6 pN (Relative *F*_*i*_ = 1), *z* = 0.4, *b* = 20 s^–1^, and *a*_*dE*_ = 0 ([P_i_] only affects the reverse rate). **A** At *t* = 0, δ*E* = kT⋅ln([P_i_]_f_/[P_i_]_i_) (Eq. 7), where [P_i_]_f_ and [P_i_]_i_ = 0.5 mM are the final and initial phosphate concentrations. [P_i_]_f_ was set to values ranging from 0.7 to 10 mM (curves top to bottom) and for each [P_i_]_f_ simulations were run (Eqs. 5 and 6) as in Fig. 2A. **B** [P_i_]_i_ was set to values ranging from 0.5 to 10 mM (curves top to bottom) and [P_i_]_f_ = [P_i_]_i_ + 1.4 mM P_i_ and simulations were run as in Fig. 2A. **C** The percent decrease in the phase 2 amplitude relative to the initial force was determined and plotted for different [P_i_]_f_ (red squares) and overlayed with corresponding experimental data (black circles) digitized from Dantzig et al. Figures 5 and 20ºC (Dantzig et al. 1992). **D** The phase 2 rate determined from single exponential fits of phase 2 in both panels A (blue squares) and B (red squares) is plotted and overlayed with experimental data (black circles) digitized from Dantzig et al. Figures 6 and 20ºC (Dantzig et al. 1992)

The myosin switch is associated with the release of P_i_ (Cooke and Pate 1985; Baker et al. 1999, 2002). Thus, a rapid increase in [P_i_] from an initial concentration, [P_i_]_i_, to a final concentration, [P_i_]_f_, increases the system free energy by δ*E* = kT⋅ln([P_i_]_f_/[P_i_]_i_). Starting from the same initial equilibrium conditions (see Fig. 5 legend) used for the length-step experiments above (only here *f_*° = 0.01 s^–1^) phase 1 is simulated simply by setting δ*E* to kT⋅ln([P_i_]_f_/[P_i_]_i_). Unlike in length step simulations (Fig. 5), force does not change with this transition, and so phase 1 is not mechanically observed.

Figures 5A and 5B show simulated chemical relaxations for different combinations of [P_i_]_f_ and [P_i_]_i_. Because *δE* in Eq. 6 does not discriminate between whether the energy change is chemical or mechanical, the same phases (phases 2 and 3) emerge from a chemical increase in *δE* as emerge from a mechanical increase in *δE* (a rapid increase in length, Δ*L*, Fig. 4A). Figure 5C is a plot of phase 2 amplitudes obtained at different [P_i_]_f_ overlaid with experimental data (Dantzig et al. 1992). Figure 5D is a plot of phase 2 rates obtained at different [P_i_]_f_ overlaid with experimental data (Dantzig et al. 1992).

## Discussion

In 1938, based on careful measurements of muscle power and heat output, A.V. Hill developed a chemical thermodynamic model of muscle contraction. At the time little was known about molecular mechanisms of muscle contraction, but A.V. Hill knew that once discovered, his model provided the framework into which the “detailed machinery must be fitted” (Hill 1966). The analysis herein further supports the hypothesis that the “detailed machinery” of Hill’s thermodynamic model is an entropic spring.

Single molecule mechanic studies directly show that the molecular mechanism of muscle contraction is a force-generating myosin motor switch, and an ensemble of molecular switches functions as an entropic spring. We have previously shown that an entropic spring accurately accounts for the relationship between muscle force and shortening velocity. Here, I have shown that an entropic spring accurately accounts for the four phases of a muscle force transient following both chemical and mechanical perturbations. This is the simplest possible model of one of the most complex mechanical behaviors of muscle.

Four well-defined thermodynamic phases of a muscle force transient emerge from a single entropic spring. Phase 1 occurs with a rapid change, *δE*, in the system energy. Phase 2 occurs when the non-equilibrium entropic spring equilibrates at a non-ergodic isotherm. Phase 3 occurs with chemical relaxation toward the ergodic isotherm. And phase 4 occurs with a return to the initial force along the ergodic isotherm. These four thermodynamic processes that correspond to four transient phases contrast with conventional molecular power stroke interpretations where a different molecular mechanism is defined for each phase.

The model parameters in Table 1 were chosen to be consistent with experimental data (both experimental measurements of the parameters as well as the experimental data simulated). A least squares fitting routing was not used in the above analysis. A minimal model is presented even though additional parameters could be justified. For example, in all simulations changes in *a* were assumed to be single exponential (Eq. 9) even though more complex force- or time-dependences of *a* are justified and could be used to significantly improve fits to experimental data. Moreover, additional biochemical states in the actin-myosin ATPase reaction cycle exist that provide additional parameters that can be used to significantly improve fits to experimental data. The bottom line is that while fits to time courses can be improved by expanding the model, the four thermodynamic phases of a force transient emerge from a single entropic spring and are consistent with the four phases experimentally observed.

## Methods

### A two state kinetic scheme

Figure 1A illustrates a simple two-state kinetic scheme (Lymn and Taylor 1971; Goldman 1987; Cooke 1997; Sweeney et al. 2020) in which a myosin (M) motor with bound ADP (D) and inorganic phosphate (P_i_) undergoes a switch-like lever arm rotation induced by actin (A) binding and gated by P_i_ release (MDP to AMD) (Huxley and Simmons 1971; Rayment et al. 1993; Finer et al. 1994; Baker et al. 1998, 2002). This molecular switch reversibly displaces a compliant element external to the motor a distance, *d*, with force-dependent forward, *f*_*+*_, and reverse, *f*_*–*_, rates (Baker et al. 2002; Stewart et al. 2021; Baker 2022). An ensemble of molecular switches is an entropic system spring (Baker 2022, 2023c).

For an equilibrium mixture of *N* parallel force generators, the displacement of a system spring by a single working step is *d*/*N* (displacing a single bed spring a distance, *d*, displaces the system of *N* parallel springs a distance *d*/*N*). At equilibrium, the system force, *F*, is distributed among all *N* myosin motors (Baker and Thomas 2000) because all motors (bound and detached) are inextricably part of and equilibrate with the macromolecular assembly to which force is applied. If only a fraction, *a*, of motors are equilibrated with the system force, the step size is *d/*(*a*·*N*), where the ergodic factor, *a*, ranges from 1 at an ergodic equilibrium to 1/*N* when on average only 1 of *N* motors is equilibrated with the system force.

### Binding free energy

The Gibbs reaction free energy for actin-myosin binding (Fig. 1A) defined at a constant muscle force, *F* (Baker et al. 1999; Baker and Thomas 2000) is

1 $${\varDelta }_{\text{r}}\text{G} = \varDelta {\text{G}}^{\text{o}} + \text{k}\text{T}\cdot\text{l}\text{n}\left[\right({N}_{AMD} + 1)/{N}_{MDP}]\hspace{0.17em}+\hspace{0.17em}F\cdot{d/aN}$$

where ∆G^o^ is the standard free energy for the binding reaction in Fig. 1A; *N*_*AMD*_ and *N*_*MDP*_ are the number of myosin motors in the AMD and MDP states (the total number of myosin motors is *N* = *N*_*AMD*_ + *N*_*MDP*_); and the logarithmic term is the change in system entropy with a chemical step from {*N*_*MDP*_, *N*_*AMD*_} to {*N*_*MDP*_–1,*N*_*AMD*_+1} along the system reaction coordinate (Fig. 1C, y-axis, right to left) (Baker 2022). Here, I approximate (*N*_*AMD*_ + 1) as *N*_*AMD*_ and assume that fixed concentrations of actin and basal inorganic phosphate, P_i_, are implicit in ∆G^o^.

### An Entropic System Spring

Here I assume a thermodynamic force exerted on an entropic system spring (Fig. 1B, right side) defines the effective stiffness, *κ*_*sys*_, of this spring (Baker 2022). I assume that one end of the spring (Fig. 1B, right) defines the macroscopic mechanical state of muscle (force, *F*, and length, *L*), while the other end of the spring (Fig. 1B, left) is stretched by force-generating switches that generate force in the spring through *κ*_*sys*_⋅*d*/(*aN*) incremental displacements. In isometric muscle (the right side of the spring in Fig. 1B is fixed) no energy is lost to the surroundings as shortening heat or work, and so I refer to isometric force generation as adiabatic. In this case force, *F*, increases linearly with the number of bound myosin motors, *N*_*AMD*_, or decreases linearly with the number of detached myosin motors, *N*_*MDP*_, as

2 $$F = \kappa_{sys}\cdot{d}/\left(aN\right)[{N}_{MDP} - {N}_{MD{P}^{\text{o}}}]$$

where *N*_*MDP*_^o^ is *N*_*MDP*_ at *F* = 0. Multiplying both sides of Eq. 2 by *d/N*

3 $$Fd/N = -a\left({\kappa }_{sys}\cdot{(d/aN)}^{2}\right)[{N}_{MDP} - {N}_{MD{P}^{\text{o}}}].$$

Equation 3 is plotted as *Fd/N* versus *N*_*MDP*_ in Fig. 1C (solid black lines at *N*_*MDP*_^o^ increments of 5).

Adiabatic (isometric) molecular force generation (Eq. 2) in the system spring continues until the force, *F*, reaches an isotherm (Eq. 1), which occurs when

4 $$Fd/N = a[\varDelta {\text{G}}^{\text{o}} + \text{k}\text{T}\cdot\text{l}\text{n}({N}_{AMD}/{N}_{MDP}\left)\right].$$

The isotherm is ergodic if *a* = 1 and is otherwise non-ergodic. Equation 4 is plotted as *Fd/N* versus *N*_*MDP*_ in Fig. 1C (colored curves with ∆G^o^ increments of 1 kT).

Equations 3 and 4 are two different definitions of system force, *F*, distinguished by whether changes in muscle force occur adiabatically (Fig. 1C, left side) or isothermally (Fig. 1C, right side). In an ideal system (adiabatic or isothermal) the binding reaction follows one or the other of these pathways (black lines or colored curves in Fig. 1C). However in general these two processes can occur together, requiring that Eqs. 3 and 4 are coupled. The physical basis for this coupling are kinetic rates that are defined by the energetics in Eq. 4 as

5 $${f}_{+}/f_{-} = \text{e}\text{x}\text{p}\left[\right(-Fd/aN - \text{k}\text{T}\cdot\text{l}\text{n}({N}_{AMD}/{N}_{MDP}) - \varDelta {\text{G}}^{\text{o}})/\text{k}\text{T}],$$

where Eq. 5 is simply the right-hand side of Eq. 1 rewritten to describe the probability of the muscle system being found in state {*N*_*MDP*_,*N*_*AMD*_} relative to {*N*_*MDP*_–1,*N*_*AMD*_+1} along a system reaction energy landscape that is tilted by the energy terms in Eq. 5 (Baker 2023c).

When *a* = 1, molecular force generation stalls along the ergodic isotherm (∆_r_G = 0) at a force, *F*_*o*_ = –N∆G^o^/*d* – NkT⋅ln(*N*_*AMD*_/*N*_*MDP*_). When *a* < 1, molecular force generation stalls along a non-ergodic (∆_r_G < 0) isotherm at a force, *F* = *a*⋅*F*_*o*_. In general, non-ergodicity occurs when not all of the *N* myosin motors equilibrate with the system force. There are many possible mechanisms for non-ergodicity, including sequestration of myosin motors in inactive states, a stiff system that generates large forces with a small number of steps, and internal system forces that contribute to the total force with which motors equilibrate.

If the internal energy of a system is perturbed by *δE*, the system energy landscape is further tilted by *δE* along the system reaction coordinate (Baker 2023c), perturbing the kinetics of force generation from Eq. 5 as

6 $${f}_{+}/{f}_{-}= \text{e}\text{x}\text{p}\left[\right(-Fd/aN - \text{k}\text{T}\cdot\text{l}\text{n}({N}_{AMD}/{N}_{MDP}) - \varDelta {\text{G}}^{\text{o}} - E)/\text{k}\text{T}].$$

For each energy term, *E*, in Eq. 6 the *E*-dependence of exp(*E*/kT) can be partitioned between forward, *f*_*+*_(*E*), and reverse, *f*_*–*_(*E*), rate constants through a coefficient, *α*_*E*_, that describes the fractional change in *E* prior to the activation energy barrier (Hille 1987). For example, when *δE* and *F* in Eq. 6 are zero,

$${f}_{+}^{\text{o}} ={N}_{MDP}\, \text{e}\text{x}\text{p}(-{\alpha}_{G}\cdot\varDelta {\text{G}}^{\text{o}}/\text{k}\text{T}) \,and$$

$${f}_{-}^{\text{o}} = {N}_{AMD} \,\text{e}\text{x}\text{p}\left[\right(1 - {\alpha}_{G})\varDelta {\text{G}}^{\text{o}}/\text{k}\text{T})$$

are unloaded equilibrium rates.

The time courses for all processes, both ideal (Eqs. 3 and 4) and non-ideal (Eqs. 3 and 4 coupled through Eq. 6), are described by three simple master equations. The rate of the two-state reaction in Fig. 1A is

7 $$\text{d}{N}_{AMD}/\text{d}\text{t}= -{N}_{AMD} \cdot{f}_{-} + {N}_{MDP}\cdot{f}_{+},$$

which according to Eq. 2 generates force in the system spring at a rate

8 $$\text{d}F/\text{d}\text{t} = (\text{d}{N}_{AMD}/\text{d}\text{t})\cdot{\kappa }_{sys}\cdot{d}/N.$$

If force generation through Eq. 8 stalls at a non-ergodic isotherm, the system subsequently approaches an ergodic isotherm at a rate *b* (i.e., the rate at which *a* approaches 1), defining the third master Eq. 

9 $$\text{d}(1-a)/\text{d}\text{t} = -(1-a)b.$$

For each non-ergodic factor that “damps” or “frustrates” equilibration (e.g., *a* < 1), there is a corresponding process (with a unique rate, *b*) through which the system reaches an ergodic equilibrium. This need not be a single exponential process. If *b* = 0, muscle is stuck in a state (e.g., smooth muscle latch) analogous to a frustrated spin state (Baker et al. 2003).

From these equations (Eqs. 7–9), four phases of a force transient are evident. The initial perturbation, *δE*, from equilibrium is phase (1) Force generation that reaches a non-ergodic isotherm (Eq. 8) is phase (2) Chemical relaxation toward the ergodic isotherm (Eq. 9) is phase (3) And a return to the original stall force along the equilibrium isotherm is phase (4) All simulations below are based on these three master equations (Eqs. 7–9) with rate constants defined from first principles (Eq. 6) and parameters defined in Table 1.

### Thermodynamic analysis

Equation 3 (black lines) and 4 (colored curves) are plotted in Fig. 1C. Equation 4 is plotted at different ∆G^o^ values (1 kT increments), and Eq. 3 is plotted at different *N*_*M*_^o^ values (increments of 5). Each line represents a reversible binding reaction, and many complex mechanical behaviors resembling muscle mechanics emerge from these binding pathways.

### Computer simulations

Using master Eqs. 7, 8 and 9, the rate constants defined by Eq. 6, and model parameters in Table 1, MatLab (Mathworks, Natick, MA) is used to simulate muscle force transients.
